# Supplementary material for: Membrane-partitioned cell wall synthesis in mycobacteria
Source: eLife. 2021 Feb 5;10:e60263. doi: 10.7554/eLife.60263 (PMC7864634; doi:10.7554/eLife.60263)
Supplement: Supplementary file 1. — (a) Incubation conditions. The table describes the incubation conditions employed in this study, including the concentrations and exposure times to a reagent. (b) Metabolic labeling conditions The table highlights the incubation conditions in which metabolic labeling was performed. (c) NCBI Accession numbers from 16S rDNA. This table provides the NCBI accession numbers of the rDNA sequences used to create the phylogenetic tree in Figure 3D. [file elife-60263-supp1.docx]

Supplementary files

**Membrane-partitioned cell wall synthesis in mycobacteria**

Alam García-Heredia^1^, Takehiro Kado^2^, Caralyn E. Sein^2^, Julia Puffal^2†^, Sarah H. Osman^2^, Julius Judd^3††^, Todd A. Gray^3,4^, Yasu S. Morita^1,2,*^, M. Sloan Siegrist^1,2,*^.

| **Supplementary file 1a. Incubation conditions** | | |
| --- | --- | --- |
| Bacterium | Antibiotic or compound | Incubation conditions |
| *M. smegmatis* | 100 mM benzyl alcohol | 60 min at 37ºC with shaking |
|  | 0.5 mM dibucaine | 180 min at 37ºC with shaking |
| DivIVA-eGFP-ID | 50 ng/mL ATC | 8 h, 14 to 16 h |
| MurJ-ID |  | 8h |
| *B. subtilis* | 24 µg/mL kanamycin | 10 min at 37°C with shaking |
|  | 50 µg/mL phosphomycin |  |
|  | 50 mM benzyl alcohol |  |
| *C. crescentus* | 5 µg/mL kanamycin | 30 min at 30°C with shaking |
|  | 50 µg/mL phosphomycin |  |
|  | 50 mM benzyl alcohol |  |
| *E. coli* | 20 µg/mL A22 | 90 min at 37°C with shaking. Cultures were back-diluted after 40 min of incubation to an OD_600_ ~0.3-0.5 in prewarmed medium + A22 and incubated until completion of 90 min |
|  | 16 µg/mL kanamycin | 30 min at 37ºC with shaking |
|  | 50 µg/mL phosphomycin |  |
|  | 50 mM benzyl alcohol |  |
| *S. aureus* and *L. lactis* | 50µg/mL phosphomycin | 30 min at 37ºC with shaking |
|  | 20 µg/mL kanamycin  50 mM benzyl alcohol |  |
|  |  |  |
|  | 50 µg/mL ampicillin (with 5 µg/mL clavulanate) |  |

| **Supplementary file 1b. Metabolic labeling conditions** | | |
| --- | --- | --- |
| Bacterium | Probe | Labeling conditions |
| *M. smegmatis* | alkDADA  N-Alk-TMM  O-Alk-TMM | Unless otherwise indicated, 15 min with shaking at 37ºC |
| *E. coli*  *B. subtilis*  *S. aureus*  *L. lactis* | alkDADA | Unless otherwise indicated, 5 min with shaking at 37ºC |
| *C. crescentus* |  | 5 min with shaking at 30ºC |

| **Supplementary file 1c. NCBI Accession numbers from 16S rDNA** | |
| --- | --- |
| Bacterium | NCBI accession number |
| *M. smegmatis* | AB305022.1 |
| *B. subtilis* | NR_112116.2 |
| *S. aureus* | NR_118997.2 |
| *L. lactis* | FJ348447.1 |
| *E. coli* | NR_024570.1 |
| *C. crescentus* | NR_037099.1 |
